# Supplementary material for: Asynchronous magnetic resonance elastography: Shear wave speed reconstruction using noise correlation of incoherent waves
Source: Magn Reson Med. 2022 Oct 27;89(3):990–1001. doi: 10.1002/mrm.29502 (PMC9792433; doi:10.1002/mrm.29502)
Supplement: Supplementary file 1 — DATA S1 MATLAB code used to generate the numerical simulations of Figure 1 [file MRM-89-990-s002.zip › k-Wave/helpfiles/example_tvsp_acoustic_field_propagator.html]

Simulating CW Fields Using The Acoustic Field Propagator Example (k-Wave)


# Simulating CW Fields Using The Acoustic Field Propagator Example

This example demonstrates how to use `acousticFieldPropagator` to simulate the steady-state pressure field from a steered line array in 2D without time stepping.

- Open the file in the MATLAB Editor
- Run the file in MATLAB

For a more detailed discussion of this example and the underlying techniques, see B. E. Treeby, J. Budisky, E. S. Wise, J. Jaros, and B. T. Cox, "Rapid calculation of acoustic fields from arbitrary continuous-wave sources," *The Journal of the Acoustical Society of America*, vol. 143, no. 1, pp.529-537, 2018.

## Contents

- Overview
- Running the simulation

## Overview

When simulating the output from time-varying pressure sources, if the driving signal is a continuous wave sinusoid and the acoustic medium is lossless and homogeneous, the steady-state acoustic field can be calculated very rapidly (without time stepping) using the function `acousticFieldPropagator`. For this function, the source is defined as two matrices of amplitude and phase which capture both the source geometry (the amplitude matrix is set to zero outside the support of the source) and the distribution of amplitude and phase across the source. In this example, the output from a steered line array is calculated in 2D. The steering angle is swept from -60 to 60 degrees with no apodisation (the amplitude is set to one across the array). For each steering angle, the phase at each grid point in the line array is calculated.

```
% assign constant amplitude across the line array
amp_in(x1, y1:y2) = 1;

% get the current steering angle
steering_angle = angle_array(index);

% calculate phase offset for each grid point in the line array based on
% element position and steering angle, and assign to the line array
phase_in(x1, y1:y2) = 2 * pi * f0 * el_pos * sind(steering_angle) / c0;
```

## Running the simulation

For each steering angle, the matrices of amplitude and phase along with the grid spacing, source frequency, and medium sound speed are passed to `acousticFieldPropagator`. This calculates the steady state acoustic field (amplitude and phase) over the same size domain as the input matrices. A plot of the calculated amplitude and phase for a steering angle of -20 degrees is shown below.

```
% compute beam pattern
[amp_out, phase_out] = acousticFieldPropagator(amp_in, phase_in, dx, f0, c0);
```

To replicate this simulation using `kspaceFirstOrder2D`, `source.p_mask` could be defined as `amp_in` with `source.p` created using `createCWSignals` along with the calculated phase offset for each steering angle. For simulations in 3D (which are conducted in an analogous fashion), the function `acousticFieldPropagatorC` can be used to reduce memory usage and compute time.
